# Enrichment Pretreatment Expands the Microbial Diversity Cultivated from Marine Sediments

**DOI:** 10.3390/microorganisms11112771

**Published:** 2023-11-15

**Authors:** Meng Wang, Ning Zheng, Xuan Li, Kun Zhao, Bin-Bin Xie

**Affiliations:** State Key Laboratory of Microbial Technology, Institute of Microbial Technology, Shandong University, Qingdao 266237, China; 202020351@mail.sdu.edu.cn (M.W.); 201912423@mail.sdu.edu.cn (N.Z.); 202212512@mail.sdu.edu.cn (X.L.); 202112574@mail.sdu.edu.cn (K.Z.)

**Keywords:** enrichment pretreatment, cultivation, chitin, cellulose, novel species

## Abstract

The majority of the microbial diversity in nature has not been recovered through cultivation. Enrichment is a classical technique widely used in the selective cultivation of specific taxa. Whether enrichment is suitable for cultivation studies that aim to recover large numbers of species remains little explored. To address this issue, we evaluated the potential of enrichment pretreatment in the cultivation of bacteria from marine sediments. Upon obtaining and classifying a total of 943 pure cultures from chitin and cellulose enrichment pretreatment systems and a control system, our results showed that species obtained using enrichment pretreatment differed greatly from those without enrichment. Multiple enrichment media and different enrichment times increased the number of cultivated species in a sample. Amplicon sequencing showed that the increased relative abundance during pretreatment contributed greatly to bacterial cultivation. The testing of degradation abilities against chitin and cellulose and the whole-genome sequencing of representative strains suggested that microorganism–microorganism interactions play roles in the expanded diversity of cultivated bacteria. This study provides new insights into the abilities of enrichment in exploring cultivable diversity and mining microbial resources.

## 1. Introduction

Cultivation is the foundation of microbiological research. Currently, the majority of microorganisms have not been cultivated, especially those from natural environments such as the ocean and soil [1,2]. Great efforts have been made to develop new media and cultivation techniques, including simulating in situ cultivation (e.g., diffusion chambers) and microfluidics-based cultivation [3,4,5,6,7,8]. Recently, the so-called “culturomics” method has increased the diversity of cultivated bacteria by employing multiple types of media and increasing the scale of cultivation [9,10]. The reverse genomics method enables the targeted cultivation of novel lineages [11]. It has also been found that sample pretreatment can help to recover species that are not cultured via classical cultivation methods alone. For example, ultraviolet irradiation pretreatment was used to increase cultivated representatives in desert-sourced samples [12], and the drying pretreatment of marine sediments could improve the cultivability of phylogenetic novel bacteria [13]. In a study of the human microbiome, the pre-incubation of intestinal samples with rumen fluid and fresh sterile stools or antibiotics helped to isolate 24 bacterial species that had never been previously found in the human intestinal flora [14].

Enrichment is a widely used pretreatment method to increase the abundance of the target groups in a sample to facilitate the subsequent cultivation of these groups or the recovery of their genome sequences [15,16,17,18]. It is often used in the cultivation of extremophilic microorganisms with slow growth rates and bacterial groups with special physiological characteristics [19,20,21], for example, hydrogen-oxidizing bacteria and nitrite-oxidizing bacteria [20,21]. Metagenomics studies have shown that the diversity of a microbial community decreases during enrichment [22,23,24,25]. Thus, it seems likely that enrichment pretreatment is not suitable for cultivating a large number of taxa due to the decreased microbial diversity in the pretreated sample. However, a study showed that enrichment helped to cultivate a large number of bacterial species, and the authors found that most species were cultivated due to the resuscitation of dormant cells, rather the enrichment of their abundance [26]. So far, the potential of enrichment to prompt the cultivation of multiple taxa from a sample and the underlying mechanisms remain largely unknown. Clarifying these issues will help to improve the cultivation of not-yet cultured microorganisms by integrating enrichment pretreatment into the cultivation systems.

Cellulose and chitin are the two most abundant polysaccharides in marine environments [27,28]. A previous genomic study has revealed that marine bacteria harboring genes for the degradation of these polysaccharides are widespread on the phylogenetic tree [29]. In this study, we developed an enrichment cultivation system for the cultivation of bacteria from intertidal surface sediments, with cellulose and chitin supplemented in the enrichment media, respectively. The aim of this study is two-fold. Firstly, this study will systematically compare the taxa cultivated with and without enrichment pretreatment to evaluate the contribution of enrichment to the cultivated diversity. Secondly, this study will monitor the community dynamics and test the degradation abilities of the obtained taxa against polysaccharides to gain insights into the underlying mechanisms for bacterial cultivation with the help of enrichment. This study provides new insights into the abilities of the classical enrichment method in bacterial cultivation and highlights the possibility of using enrichment as a pretreatment method to promote the cultivation of previously uncultivated diversity.

## 2. Materials and Methods

### 2.1. Sample Collection

Three intertidal sediment samples (A, B, C) were collected at Qingdao, Shandong Province (A: 36°20′ N, 120°40′ E; B: 36°22′ N, 120°42′ E; C: 36°23′ N, 120°42′ E), in December 2020. Surface sediment samples (0–2 cm) were collected using a sterilized steel spoon at low tide, with the sampling sites about 100 m from the shoreline. The temperature of the sediments was measured using a thermometer, and the temperatures of samples A, B, and C were 5.6 °C, 5.8 °C, and 5.6 °C, respectively. The samples were stored in 50 mL sterilized centrifuge tubes and transported to the laboratory for processing within 1 h.

### 2.2. Bacterial Cultivation

#### 2.2.1. Direct Cultivation without Enrichment Pretreatment

Three grams of sediment sample was placed in a sterile 50 mL centrifuge tube, and 27 mL of sterilized sodium chloride solution (2.5%) was added. The solution was shaken manually for 15 min, and left to set for 15 min. The supernatant was considered the stock solution and serial dilutions were prepared using 2.5% sterile sodium chloride solution. The stock solution, diluted 5-fold and 10-fold, was spread onto the chitin solid medium, cellulose solid medium, and comprehensive solid medium (Table S1), respectively, with an inoculum of 100 μL per plate and three replicates per gradient (5-fold and 10-fold), and incubated at 20 °C for three weeks. The comprehensive medium is a medium developed in our laboratory that contains a variety of monosaccharides, oligosaccharides, and sugar metabolites in low concentrations, and this medium was designed to meet the nutrient requirements of as many microorganisms as possible (Table S1).

#### 2.2.2. Cultivation with Enrichment Pretreatment

The stock solution (2 mL) prepared in Section 2.2.1 was added to 100 mL sterilized chitin liquid medium and cellulose liquid medium with 2% inoculum, respectively (Table S1). After inoculation, the conical flasks were incubated in a shaker (20 °C, 200 rpm) for 92 days. During enrichment, the cultures were sampled on days 7, 14, 21, 28, 57, and 92, respectively, for further cultivation via the spread plate method. For the chitin enrichment pretreatment, the chitin solid medium and comprehensive solid medium were used in the cultivation. For the cellulose enrichment pretreatment, the cellulose solid medium and comprehensive solid medium were used in the cultivation. The plates were incubated at 20 °C for three weeks.

### 2.3. Bacterial Isolation and Identification

Colonies were picked according to their morphology, color, and size to maximize the diversity, and then, purified using the streak plate method. The *16S rRNA* gene was amplified using the primers 27F (5’-AGA GTT TGA TCM TGG CTC AG-3’) and 1492R (5’-TAC GGY TAC CTT GTT ACG ACT T-3’) [30]. The PCR products were confirmed via 1% agarose gel electrophoresis and sequenced (Sanger) at Tsingke Biotechnology Co., Ltd. (Beijing, China). The *16S rRNA* gene sequences were clustered using mothur (v 1.45.2) with 98% identity to generate OTUs [31], and each OTU was considered a species. The representative *16S rRNA* gene sequence of each OTU was submitted to the EzBioCloud server (https://www.ezbiocloud.net/ accessed on 16 April 2023) for comparison [32]. If the *16S rRNA* gene sequence was less than 98% similar to the top hit type strain, the strain was considered a novel candidate species [33].

To show the overall diversity of the cultured bacteria, the representative sequences of all species were aligned with MAFFT (v 7.450), and a phylogenetic tree was reconstructed using MEGA (v 10.0.5) via the neighbor-joining method and maximum composite likelihood model [34,35]. One thousand bootstrap replicates were generated. The tree was visualized using the iToL website (https://itol.embl.de/ accessed on 7 May 2023) [36].

### 2.4. Amplicon Sequencing and Analyses

Genomic DNA was extracted from intertidal sediments (A, B, C) as well as 7-day chitin enrichment pretreatment suspension (7A, 7B, 7C) and 92-day chitin enrichment pretreatment suspension (92A, 92B, 92C) using an E.Z.N.A soil DNA kit (Omega BIO-TEK, Norcross, GA, USA). Amplification was performed using *16S rRNA* gene V3-V4-region primers (341F: CCT AYG GGR BGC ASC AG, 806R: GGA CTA CNN GGG TAT CTA AT) [37] and sequenced using the Illumina NovaSeq 6000 platform, yielding 250 bp paired-end reads.

Low-quality sequences in raw data were filtered out using Fastp (v 0.23.1) (default parameters) [38], and paired sequences were merged using Vsearch (v 2.18.0) [39]. Sequences were clustered with 97% similarity to generate OTUs using Vsearch (v 2.18.0), chimeras were removed using the Silva database (v 123) as reference, and OTU annotation was performed with RDP (v 18) as the reference database. Alpha diversity was calculated using the vegan package in the software R (v 4.1.1). Principal coordinate analysis was conducted and visualized using the vegan and ggplot2 packages in R (v 4.1.1). To detect the relative abundance of the isolated species for each enrichment period, the representative *16S rRNA* gene sequences of isolated species were mapped to the OTU sequences of amplicon sequencing data using Vsearch (v 2.18.0) with 97% sequence identity.

### 2.5. Classification Based on the 16S rRNA Gene

To perform taxonomic classification for each novel candidate species, the *16S rRNA* gene sequences of the closely related type strains were downloaded from the EzBioCloud database. The sequences were aligned using MAFFT (v 7.450) [34], and a phylogenetic tree was reconstructed using the software MEGA (v 10.0.5) via the maximum likelihood method and maximum composite likelihood model [35]. One thousand bootstrap replicates were generated.

### 2.6. Testing of Cellulose and Chitin-Degradation Ability

Three grams of chitin powder was added to 150 mL of 37% hydrochloric acid solution and placed at 4 °C for 24 h. The precipitate was obtained via centrifugation at 9000 rpm for 10 min, and the precipitate was repeatedly dissolved with distilled water and centrifuged until the pH of the supernatant was neutral. The supernatant was removed and the precipitate was retained and stored at 4 °C. A total of 30 mL of distilled water was added to the precipitate to obtain the colloidal chitin [40], and then, the colloidal chitin was added to 270 mL of 2216E medium to prepare the colloidal chitin agar plates (autoclaving, 121 °C, 30 min). The novel strains were inoculated on the colloidal chitin agar plates and incubated at 20 °C for 15 days. A transparent circle around the colony indicated that the strain could degrade chitin [40].

To test the cellulase activity, novel candidate species were inoculated onto the carboxymethylcellulose agar plates and incubated at 20 °C for 15 days. A total of 15 mL of Congo red solution was added to the carboxymethylcellulose agar plates and stained for 20 min. Then, the plates were soaked in 1 mol/L sodium chloride solution for 15 min. After the sodium chloride solution was poured, the plates were rinsed once more with sodium chloride solution. The appearance of uncolored degradation circles around the colony indicated that the strain had cellulose degradation capacity [41].

### 2.7. Genome Sequencing and Analyses

Genomic DNA was extracted using the SDS-CTAB method [42]. A sequencing library with an average insert size of approximately 350 bp was prepared and sequenced on the Illumina NovaSeq platform. A total of 858–1612 Mbp clean data (paired-end, read length 150 bp × 2) were generated for each strain. Genomes were assembled using SOAP denovo (v 2.04), and Gapcloser (v 1.12) was used to close the gaps [43]. Genome annotations were performed using the RAST server (https://rast.nmpdr.org/ accessed on 7 July 2023) [44].

To perform phylogenomic analyses, genomes of closely related strains reported by the EzBioCloud server were downloaded from the National Center for Biotechnology Information nucleotide database. Genomes of more distantly related species were randomly selected and downloaded as outgroups. Homologous protein families were predicted using the software OrthoFinder (v 2.0), and a phylogenetic tree was reconstructed based on the single-copy shared homologous proteins using the neighbor-joining method implemented in the software MEGA (v 10.0.5) [35,45]. The average nucleotide identity (ANI) was calculated using the JspeciesWS website (http://jspecies.ribohost.com/jspeciesws accessed on 8 July 2023) [46].

### 2.8. Statistical Analyses

Statistical analyses were performed using R. For data following a normal distribution, an independent-samples t test was performed. For data not following a normal distribution, the Mann–Whitney test was performed. A *p*-value < 0.05 was considered statistically significant.

### 2.9. Data Availability

The *16S rRNA* gene sequences of representative species are available in the NCBI GenBank database under accession numbers OR078484–OR078517. The amplicon sequencing data have been deposited in the Genome Sequence Archive (GSA) of the National Genomics Data Center, China National Center for Bioinformation (CNCB-NGDC), under accession number CRA011269 (https://ngdc.cncb.ac.cn/gsa/ accessed on 2 June 2023). The whole-genome sequences have been deposited in the Genome Warehouse of the National Genomics Data Center, China National Center for Bioinformation (CNCB-NGDC), under accession numbers GWHCBJD00000000 (4AJ11), GWHCBJC00000000 (28AX09), GWHBRAG00000000 (57AJ16), GWHCBJB00000000 (57CJ19), and GWHCBJA00000000 (92AX17) (https://ngdc.cncb.ac.cn/gwh/ accessed on 2 June 2023).

## 3. Results

### 3.1. Large Difference in Diversity Cultivated with and without Enrichment Pretreatment

**Figure 1:**
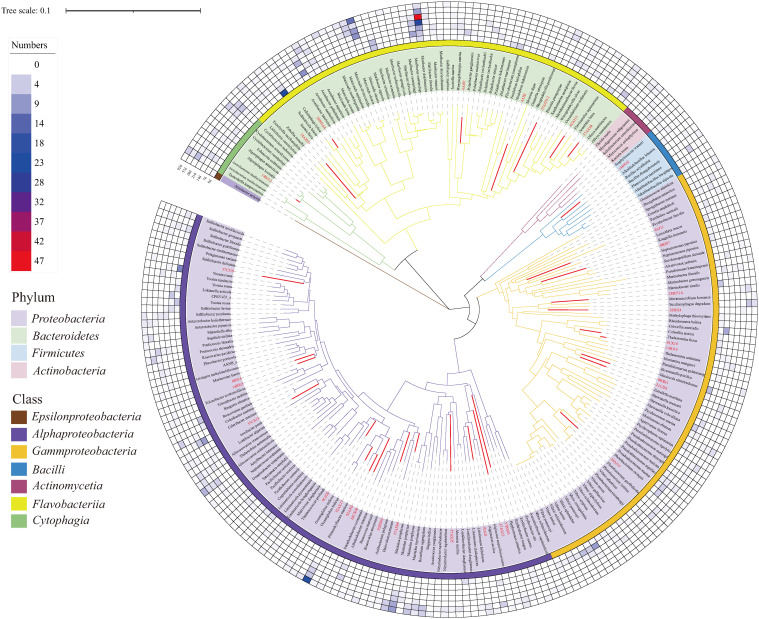
Phylogenetic tree of bacterial species cultivated from the three intertidal sediment samples. Red branches and labels represent novel bacterial species. The colors of the heatmap represent the number of strains obtained for each species. 0d indicates the bacterial strains cultivated without enrichment; 7d, 14d, 21d, 28d, 57d, and 92d indicate strains cultivated after 7-, 14-, 21-, 28-, 57- and 92-day enrichment pretreatment, respectively.

Three intertidal surface sediment samples were collected and cultivated via the spread plate method. For each sample, two pretreatment methods were used; one was enrichment in the chitin liquid medium for three months before plating, and the other was enrichment in the cellulose liquid medium for three months. Direct plating without enrichment pretreatment was used as a control. For the control without pretreatment, a total of 153 strains were selected for *16S rRNA* gene sequencing, and taxonomic classification based on the EzBioCloud server revealed that these strains belonged to 48 genera and 79 species, and the dominant genera were *Sediminicola*, *Yoonia*, *Maribacter*, *Sulfitobacter*, and *Vibrio* (Figure 1, Table S2). For enrichment pretreatment, samples were obtained from the enrichment system at six time points (7, 14, 21, 28, 57, and 92 days) for subsequent cultivation via the spread plate method. A total of 425 strains were picked from the cellulose enrichment medium, and taxonomic classification showed that these bacteria belonged to 122 species from 83 genera, with the dominant genera being *Maribacter*, *Celeribacter*, *Maritalea*, *Vibrio*, and *Primorskyibacter* (Figure 1, Table S2). For the chitin enrichment medium, 365 strains were picked, belonging to 128 species from 80 genera, with the dominant genera being *Maribacter*, *Arenibacter*, *Muricauda*, *Aliiroseovarius*, and *Cyclobacterium* (Figure 1, Table S2). On average, 9-27 species were obtained at each time point (Figure S1). In summary, a total of 943 strains were sequenced in this study, belonging to 4 phyla (*Proteobacteria*, *Bacteroidetes*, *Firmicutes* and *Actinobacteria*), 139 genera, and 248 species (Table S3).

**Figure 2:**
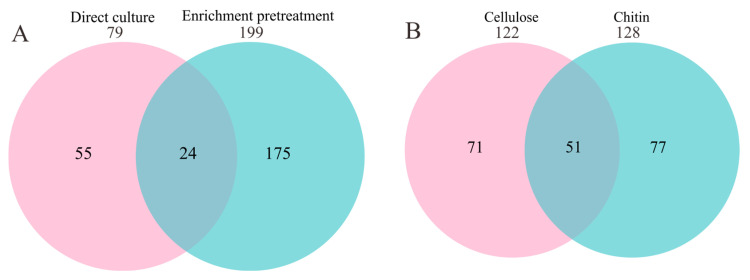
Venn diagram of species cultured with and without enrichment. (**A**) Comparison of species cultivated with and without enrichment. (**B**) Comparison of species cultivated with the cellulose and chitin enrichment media.

Next, we compared the bacterial species obtained with and without enrichment pretreatment. As shown in Figure 2A, among the total of 199 bacterial species obtained after enrichment pretreatment, 175 (88%) were not obtained without enrichment. Among the 79 species obtained by direct cultivation, 55 (70%) were not obtained after enrichment. These results indicate that enrichment pretreatment altered the diversity of the cultivated bacteria, and thus, can be used to recover species that are not recovered via the spread plate method alone. In addition, the enrichment media greatly affected the diversity of the species recovered. Among the 199 species obtained using the two enrichment media, only 51 were obtained using both enrichment media (Figure 2B).

### 3.2. Different Novel Species Recovered at Different Time Points of Enrichment

**Figure 3:**
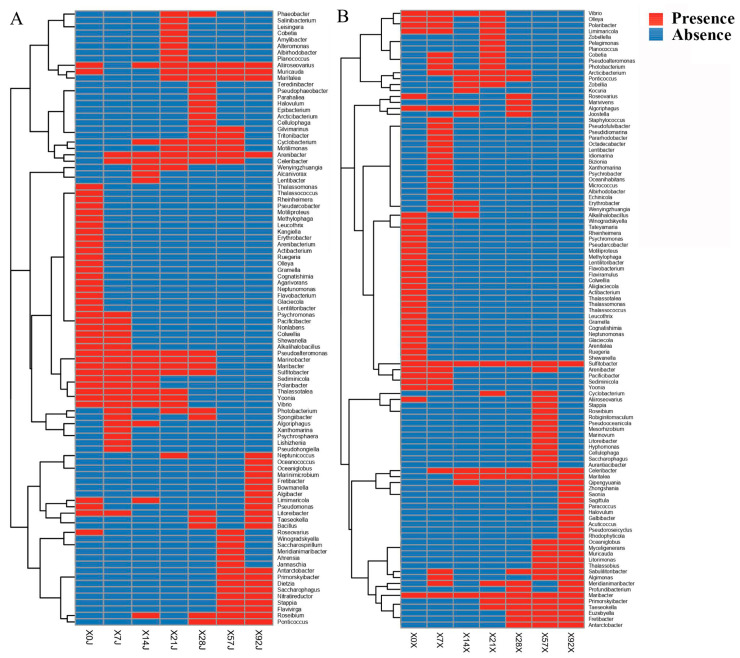
The bacterial genera obtained at different enrichment pretreatment time points. (**A**) Chitin enrichment. (**B**) Cellulose enrichment. Red and blue represent presence and absence of the genus, respectively.

**Figure 4:**
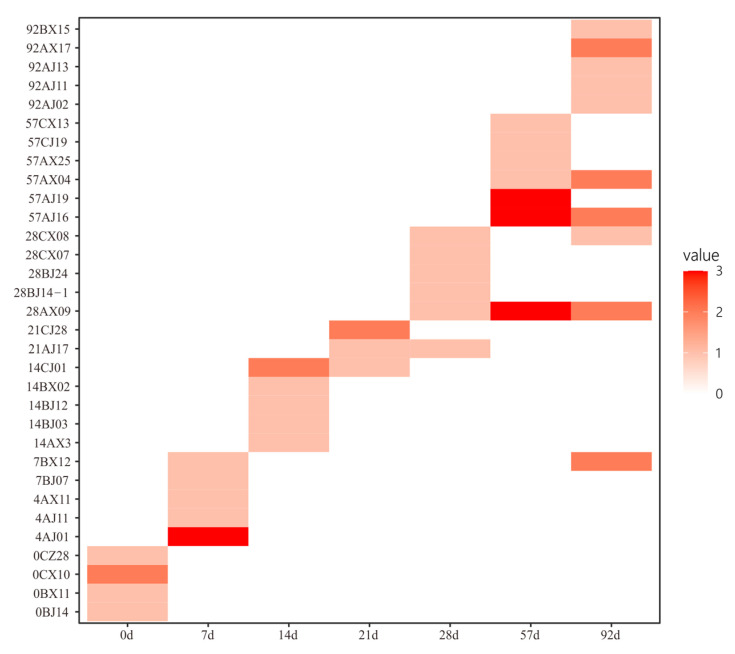
Heatmap of the novel species isolated at different enrichment pretreatment time points. The color represents the number of bacterial strains.

We compared the diversity of the bacteria obtained at different time points during enrichment and found that there were great differences among time points (Figure 3), suggesting that the cultivated bacterial community changed greatly during the enrichment pretreatment. Among the total 248 species identified in this study, 32 were novel candidate species based on the *16S rRNA* gene identity cutoff of 98% for species delineation (Table S4) [33]. These novel species belonged to 3 phyla (*Proteobacteria*, 22; *Bacteroidetes*, 9; *Firmicutes*, 1), 5 orders (*Alphaproteobacteria*, 15; *Flavobacteriia*, 8; *Gammaproteobacteria*, 7; *Bacilli*, 1; *Cytophagia*, 1), and 30 genera. Among them, strain 28AX09 showed the highest identity (92.17%) with *Fretibacter rubidus* JC2236^T^ (JQ965646), indicating that it was a novel candidate genus based on the commonly used cutoff for genus delineation of 94.5% (Table S4) [47]. Among all 32 novel candidate species, four were obtained without enrichment pretreatment and 28 were obtained after enrichment (Figure 4). Only one novel candidate species (strain 4AJ01) was found from both enrichment media (Table S4). It was noted that most of these novel candidate species were obtained at different time points during enrichment, and only a few novel species were shared by more than one time point (seven species, 22%) (Figure 4). These results indicated that sampling at different time points of enrichment pretreatment increased the diversity of the obtained novel candidate species.

### 3.3. Dynamics of Bacterial Community in the Chitin Enrichment Medium

**Figure 5:**
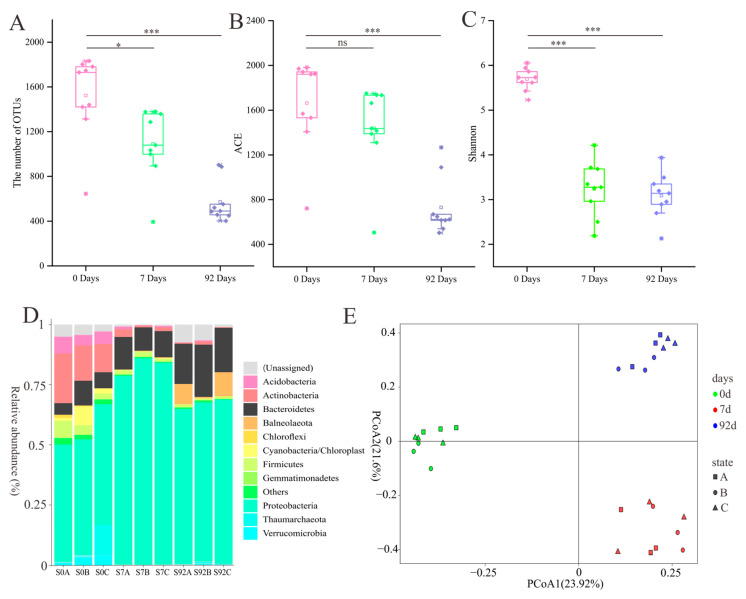
Diversity of bacterial communities after 0-day (non-enrichment), 7-day, and 92-day enrichment pretreatment with chitin medium. (**A**) The number of OTUs. (**B**) ACE index. (**C**) Shannon index. (**D**). Composition at phylum level. (**E**) Principal coordinate analysis (PCoA). ns represents *p* > 0.05, * represents *p* < 0.05, *** represents *p* < 0.001.

To reveal the changes in microbial community composition during enrichment pretreatment, pretreated samples (7 days and 92 days) and non-pretreated samples (0 day) were subject to amplicon sequencing. A total of 2,322,424 sequences were generated, and clustering produced 3644 OTUs, belonging to 41 phyla, 74 classes, 107 orders, 240 families, and 577 genera. *Proteobacteria*, *Bacteroidetes*, *Firmicutes*, and *Actinobacteria* were the dominant phyla (Figure 5D). The OTU number, ACE, and Shannon indices of the pretreated samples were significantly lower than those of the non-pretreated samples, especially after 92d (Figure 5A–C). Principal coordinate analysis showed that there was a significant difference in the microbial community compositions between the pretreated and non-pretreated samples (Figure 5E, Table S5). Samples were clustered according to the time points of enrichment, rather than the sampling site (Figure 5E).

### 3.4. Increased Relative Abundance of Cultivated Species during Enrichment

**Figure 6:**
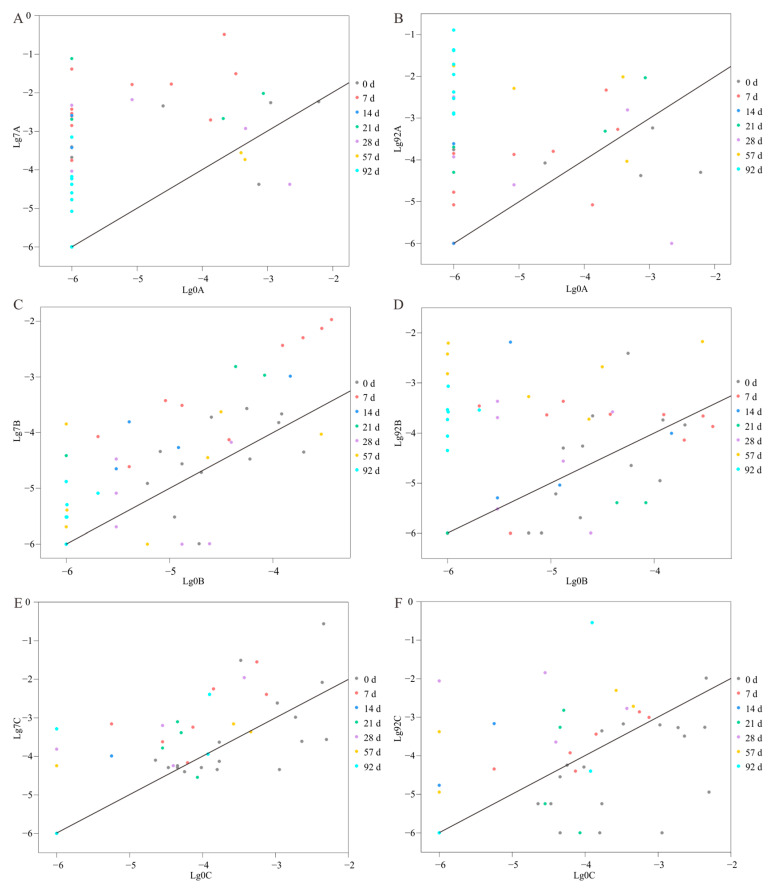
Changes in the relative abundance of cultivated species in different samples after chitin enrichment pretreatment compared with non-enrichment. (**A**) Sample A after 7-day enrichment. (**B**) Sample A after 92-day enrichment. (**C**) Sample B after 7-day enrichment. (**D**) Sample B after 92-day enrichment. (**E**) Sample C after 7-day enrichment. (**F**) Sample C after 92-day enrichment. The relative abundance is taken as a logarithmic number with a base of 10 [Log_10_(the relative abundance)]. The horizontal coordinate is the relative abundance of the cultivated species in the non-enriched sediment sample, and the vertical coordinate is the relative abundance of the cultivated species after 7-day (**A**,**C**,**E**) or 92-day (**B**,**D**,**F**) enrichment pretreatment. Each dot represents a cultivated species that can be detected via the amplicon sequencing method. Different colors represent the bacterial species isolated at different enrichment pretreatment time points.

Out of the 128 species cultivated with chitin enrichment, 79 were detected via amplicon sequencing. The total relative abundance of these 79 species was 3.6% (±2.0%, standard deviation, *n* = 9) in the non-pretreated communities, 67% (±4.0%, *n* = 9) in the 7-day pretreatment communities, and 46.9% (±6.2%, *n* = 9) in the 92-day pretreatment communities (Figure S2). These results indicate a great increase in the total abundance of the cultivated species. Moreover, among these 79 detected species, 9 were not detected in the non-pretreated communities but were detected after enrichment pretreatment (Figure S3). We also checked the relative abundance of each species in the 7-day enrichment samples. For all species that were cultivated after 7-day enrichment, their relative abundance was increased in all 7-day enrichment samples compared with the non-enriched samples (Figure 6). Similarly, 81% of the species cultivated after 92-day enrichment showed increased relative abundance in the 92-day enrichment samples compared with in the non-enriched samples (Figure 6). Therefore, the increased relative abundance during pretreatment contributed greatly to the cultivation of these bacteria.

### 3.5. Genome Sequencing and Phylogenomic Analyses of Novel Species

The degradation abilities of the 32 novel candidate species towards cellulose and chitin were tested. Surprisingly, only three of them could form degradation circles on the carboxymethylcellulose plates (0CZ28, 7BX12, and 14BJ12), and only one could form degradation circles on the colloidal chitin plates (57AJ16) (Table S4). These results indicate that most novel species were not able to degrade the polysaccharides supplemented in the enrichment media.

To gain further insights into the metabolic potential of the novel candidate species, five strains (4AJ11, 28AX09, 57AJ16, 57CJ19, and 92AX17) with the lowest *16S rRNA* gene identity to known species were selected for whole-genome sequencing. The general features of these six genomes are shown in Table 1. Consistent with the above results that only 57AJ16 showed chitin-degradation ability, genome annotation revealed that, among those sequenced, only the genome of strain 57AJ16 contained the chitinase gene (EC 3.2.1.14). It was noted that genes for enzymes involved in the metabolism of chitin degradation products, such as beta-N-acetylglucosaminidase (EC 3.2.1.52), N-acetylglucosamine-6-phosphate deacetylase (EC 3.5.1.25), and glucosamine-6-phosphate deaminase (EC 3.5.99.6), were found in the genomes of strains 4AJ11 and 57CJ19, which showed no chitin-degradation ability. These results imply the presence of cross-feeding relationships in the enrichment pretreatment system.

**Figure 7:**
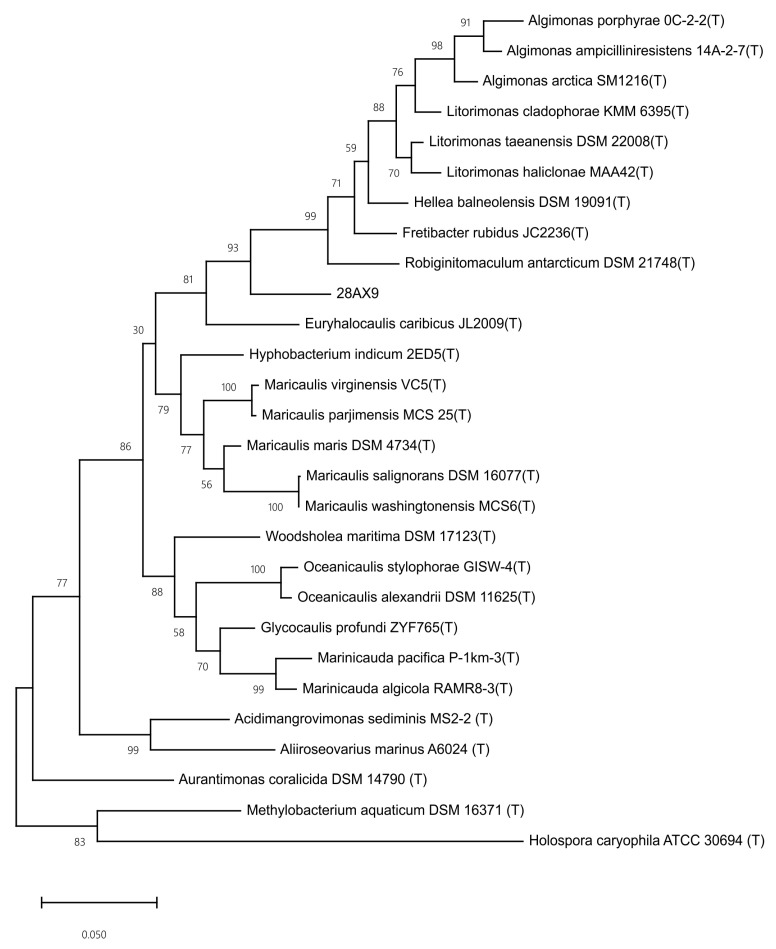
The phylogenetic tree of strain 28AX09 based on the *16S rRNA* gene sequence. The number on the branch represents the bootstrap percentage.

The taxonomic classification of the sequenced strains was determined based on the genomic analyses. The ANIs of these five strains with their close relatives were all lower than the cutoff of 95% widely used in species delineation (Table S6) [33], suggesting that they represent novel species. Phylogenetic analyses based on *16S rRNA* gene sequences and phylogenomic analyses based on homologous proteins suggested that strains 57AJ16, 4AJ11, 57CJ19, and 92AX17 represented novel candidate species within the genera *Flavivirga*, *Pseudohongiella*, *Jannaschia*, and *Rhodobacteraceae*, respectively (Figures S4–S11). Strain 28AX09 represented a novel candidate genus within the family *Robiginitomaculaceae* (Figure 7 and Figure S12).

## 4. Discussion

In this study, we pretreated intertidal sediment samples before cultivation using two different enrichment media, and found that there were great differences between the species obtained with and without enrichment pretreatment. The results also showed that different enrichment systems led to the recovery of different species. These observations are consistent with a previous report stating that microbial communities in enrichment systems are regulated by substrate types [48]. Additionally, sampling at different times during enrichment also led to the recovery of different species. Thus, similar to the multiple cultivation media and conditions used in culturomics studies [9,10,49,50], multiple enrichment media and different enrichment times can be used in the future to fully explore the microbial resources in a sample.

Enrichment is widely used to increase the relative abundance of target taxa in the community by using media containing nutrients that could be utilized by the target taxa. Rodrigues-Oliveira et al. succeeded in increasing the relative abundance of *Asgard archaeon* Loki-B35 from an initial abundance 0.03% to 79% through several enrichment cultivations [15]. In another study, after 14-month incubation while continuously adding methane, ammonium, and nitrite to the enrichment system, a stable enrichment culture dominated by two types of *Methylomirabilis oxyfera* and two strains of anammox bacteria was obtained [51]. Furthermore, a number of uncultivated microorganisms have been isolated with the help of enrichment. For example, strain L21-Fru-ABT, the first cultivated representative of *Verrucomicrobia* subdivision 5, was cultivated from an anoxic sample of a cyanobacterial mat [16]. Strain RT761, a member of the candidate phylum “*Atribacteria*”, was isolated after 3 years of enrichment from saline formation water and sediments [18]. As revealed via amplicon sequencing in this study and other studies, the richness of the community decreased during enrichment [22,23,24,25], which is not favorable for the cultivation of a high diversity of bacteria. Even so, up to 199 species were cultivated after enrichment, indicating high richness of the community even after enrichment. Moreover, besides members that could directly utilize the nutrients in the enrichment media, other community members may also be cultivated. In this study, most strains representing novel candidate species lacked cellulose/chitin-degrading abilities. Further genomic analyses suggested that some strains could utilize the chitin degradation products released by the chitin-degrading taxa. Thus, the high richness of the community may be partially maintained by the potential interactions between different groups of bacteria, for example, the interaction between chitin degraders and other bacteria utilizing the chitin degrading products released by the chitin degraders.

The majority of marine microbes have not been cultured under laboratory conditions (>99%) [4]. To cultivate novel microbial species, a series of new cultivation strategies have been developed, such as those based on cell-directed isolation strategies (e.g., reverse genomics, fluorescence-activated cell sorting, and microfluidics cultivation) [8,11], in situ cultivation strategies based on simulated natural environments (e.g., diffusion chamber, ichip, and trap) [7,52,53], and co-cultivation strategies based on cell interactions [54]. These new strategies have played important roles in the discovery of novel taxa, but the instrumentation dependence (e.g., microfluidic devices, flow cytometry) and experimental complexity have constrained their widespread use. Enrichment culture is a simple and effective method for the targeted cultivation of microorganisms for specific functions. A recent study showed that the combination of enrichment pretreatment with new culture strategies can also accelerate the cultivation of novel taxa [55]. In that study, the combined use of enrichment cultivation and a double emulsion platform successfully enabled the cultivation of slower-growing microbes, such as *Negativicutes* and *Methanobacteria* [55]. Here, in this study, we cultivated 34 novel species through a combination of the enrichment pretreatment and the traditional cultivation technique, demonstrating that the simple enrichment approach is effective in novel species discovery. The following properties of enrichment pretreatment may contribute to the discovery of novel species. Firstly, enrichment can awaken dormant cells in the in situ sample and increase the abundance of some low-abundance species [24,26], allowing access to species that are difficult to cultivate via the spread plate method. Secondly, the enrichment system is a mixed system that preserves cooperation or coordination among microbial species. Finally, prolonging the enrichment pretreatment time can leave sufficient time for slow-growing taxa to proliferate.

Among the novel species recovered, only strain 57AJ16 could form transparent degradation circles on colloidal chitin plates, and the gene encoding chitinase was found in its genome. To our knowledge, this is the first bacterium of the genus *Flavivirga* (belonging to the family *Flavobacteriaceae*) found to degrade chitin. Many bacteria within the family *Flavobacteriaceae* can degrade chitin, such as *Flavobacterium johnsoniae* [56], which is best known for its ability to rapidly digest insoluble chitin [56], and *Aquimarina hainanensis*, a strain isolated from diseased mud crab *Scylla serrata* larvae [57]. Notably, most of the novel bacteria do not directly degrade chitin, but a series of genes for enzymes involved in the metabolism of chitin degradation products were found in some non-chitin-degrading bacteria, such as *Jannaschia* sp. 57CJ19 and *Rhodophyticola* sp. 92AX17, and these bacteria can be considered “chitin degradation products utilizers”. In addition, the byproducts produced by chitin cross-feeders can also benefit other microorganisms that are not involved in chitin degradation. A study has revealed that *Alteromonas macleodii* can benefit from the by-products of chitin-degrading bacteria, even though *Alteromonas macleodii* cannot degrade chitin and consume chitin degradation products [58]. Coincidentally, the bacterial strain *Alteromonas* sp. 21CJ28 was cultivated in our chitin pretreatment system, but cannot degrade chitin. Among the novel species, three species have cellulose-degrading ability (*Thalassococcus* sp. 0CZ28, *Algibacter* sp. 7BX12, and *Algoriphagus* sp. 14BJ12). It has been reported that members of the genus *Algibacter* have the ability to degrade cellulose [59]. Studies have shown that the genus *Algoriphagus* was significantly enriched in wheat straw (or rice straw)-degrading systems [60,61], implying that this genus may be involved in cellulose degradation. Cellulases can degrade cellulose into cellodextrin, cellobiose, D-glucose, and other products that can be easily utilized by organisms. Most of the novel bacteria cultivated from cellulose pretreatment system, such as *Pseudofulvibacter* sp. 4AX11, *Psychroserpens* sp. 14AX03, and *Maribacter* sp. 28CX07, cannot degrade cellulose, but most of the bacteria from the above genera can utilize cellobiose and D-glucose [62,63,64]. Therefore, these bacteria can be considered beneficiaries of cellulose degradation, which may also be the reason why they were cultivated.

In this study, amplicon sequencing revealed that the majority of bacterial species cultivated from the chitin enrichment pretreatment system showed increased relative abundance after pretreatment, suggesting that increased relative abundance (enrichment) is an important mechanism in the cultivation of these bacteria. This observation is consistent with the many studies in which the relative abundance was increased in the enrichment system [65,66,67]. A previous enrichment study showed a different result, that is, the relative abundance of 80% of the species cultivated after enrichment was not increased during enrichment [26]. Many factors may contribute to this difference. For example, the above study used an interesting enrichment cultivation system, in which the medium for cultivation was different from that for enrichment, and therefore, the dominant species enriched by the pretreatment may not be cultivated in the subsequent cultivation.

The composition of the microbial community changed significantly during enrichment pretreatment. The relative abundance of *Bacteroidetes* and *Balneolaeota* increased, and *Acidobacteria*, *Verrucomicrobia*, and *Thaumarchaeota* disappeared or decreased in relative abundance. *Bacteroidetes* is famous for its ability to degrade a wide range of complex carbohydrates [68,69]. An important feature of the *Bacteroidetes* genome is the presence of polysaccharide utilization loci [68], and this trait may contribute to the increase in its relative abundance in the pretreatment system. *Balneolaeota* is a new phylum separated from *Bacteroidetes* [70,71]. Although the physiological characteristics of this phylum are poorly understood, many genes involved in carbohydrate transport and metabolism have been identified in their genomes [72]. Bacteria of *Acidobacteria* are generally oligotrophic and difficult to cultivate [73], and several studies have revealed that the relative abundance of *Acidobacteria* declines significantly as the nutrient content of the soil is elevated [74,75]. Compared to the marine environments, the enrichment pretreatment system provided rich nutrients, and this may contribute to the decline in the relative abundance of *Acidobacteria*. Interestingly, several studies found that many members of *Verrucomicrobia* are extensively involved in polysaccharide degradation [76], but *Verrucomicrobia* decreased in relative abundance in our enrichment pretreatment system. Studies on the cultivation of *Verrucomicrobia* showed that *Verrucomicrobia* grows slowly and requires either extended cultivation times or the addition of antibiotics to the medium to inhibit the growth of highly abundant bacteria to successfully obtain pure cultures [77,78]. Therefore, *Verrucomicrobia* may proliferate more slowly than other bacteria that can utilize polysaccharides (e.g., *Bacteroidetes*), causing a decrease in relative abundance. Similarly, the growth rate of archaea is slow [79], and the relative abundance of the *Thaumarchaeota* in the enrichment pretreatment system was decreased.

## 5. Conclusions

In this study, we cultivated 248 species from intertidal surface sediments and demonstrated that the enrichment pretreatment substantially altered the diversity of the cultivated bacteria compared with the spread plate method alone. Our results suggest that a combination of multiple enrichment media and different enrichment times will help to recover the unexplored diversity of cultivable microorganisms. This study provides new insights into the potential of the classical enrichment method in the cultivation of the previously uncultured majority.

## Figures and Tables

**Table 1:** Overview of the genomes of five novel strains.

Bacterial Strains	Size (bp)	N50 Len. (bp)	GC Content (%)	Number of Coding Sequences	Genes Related to the Metabolism of Cellulose and Chitin and Their Degradation Products
4AJ11	3,177,342	1,663,422	49.3	2881	beta-N-acetylglucosaminidase (EC 3.2.1.52)
28AX09	3,151,738	1,843,572	56.9	2906	endoglucanase, beta-glucosidase (EC 3.2.1.21), beta-N-acetylglucosaminidase (EC 3.2.1.52), beta-glycosyl hydrolase
57AJ16	4,920,416	227,616	34.3	4385	endoglucanase beta-glucosidase (EC 3.2.1.21) chitinase (EC 3.2.1.14) beta-glycosyl hydrolase N-acetylmuramic acid 6-phosphate etherase (EC 4.2.1.126) N-acetyl glucosamine transporter (NagP) N-acetylglucosamine related transporter (NagX) Glucosamine-6-phosphate deaminase (EC 3.5.99.6) beta-glycosyl hydrolase Glucosamine-6-phosphate deaminase (EC 3.5.99.6)
57CJ19	3,915,335	2,086,907	61.2	3913	beta-glucosidase (EC 3.2.1.21) beta-N-acetylglucosaminidase (EC 3.2.1.52) N-acetylmuramic acid 6-phosphate etherase (EC 4.2.1.126) Glucosamine-6-phosphate deaminase (EC 3.5.99.6) N-acetylglucosamine kinase of eukaryotic type (EC 2.7.1.59) N-acetylglucosamine-6-phosphate deacetylase (EC 3.5.1.25) Glucosamine-6-phosphate deaminase (EC 3.5.99.6)
92AX17	3,864,317	1,137,868	66.6	3809	beta-glucosidase (EC 3.2.1.21) beta-N-acetylglucosaminidase (EC 3.2.1.52) Glucosamine-6-phosphate deaminase (EC 3.5.99.6) N-acetylglucosamine-6-phosphate deacetylase (EC 3.5.1.25) Glucosamine-6-phosphate deaminase (EC 3.5.99.6)

## Data Availability

The *16S rRNA* gene sequences of representative species are available in the NCBI GenBank database under accession numbers OR078484–OR078517. The amplicon sequencing data have been deposited in the Genome Sequence Archive (GSA) of the National Genomics Data Center, China National Center for Bioinformation (CNCB-NGDC), under accession number CRA011269 (https://ngdc.cncb.ac.cn/gsa/ accessed on 2 June 2023). The whole-genome sequences have been deposited in the Genome Warehouse of National Genomics Data Center, China National Center for Bioinformation (CNCB-NGDC), under accession numbers GWHCBJD00000000 (4AJ11), GWHCBJC00000000 (28AX09), GWHBRAG00000000 (57AJ16), GWHCBJB00000000 (57CJ19), and GWHCBJA00000000 (92AX17) (https://ngdc.cncb.ac.cn/gwh/ accessed on 2 June 2023).

## Supplementary Materials

The following supporting information can be downloaded at: https://www.mdpi.com/article/10.3390/microorganisms11112771/s1, Figure S1: The number of species obtained at different enrichment time points. (A). Chitin enrichment pretreatment, (B) cellulose enrichment pretreatment. The bar chart represents the number of cultivated bacterial species at each enrichment pretreatment time point. Error bars are based on standard deviation (*n* = 3). The line is the accumulation curve of bacterial species obtained during the enrichment pretreatment. Figure S2: The sum of relative abundance of 79 cultivated species detected via amplicon sequencing in the in situ (non-enriched), 7-day, and 92-day enrichment pretreatment communities, respectively. Error bars are based on standard deviation. Figure S3: The relative abundance of each strain in the community (left) and the origin of the strain (right). A-0d, B-0d, and C-0d indicate in situ (non-enriched) samples A, B, and C, respectively. A-Eri, B-Eri, and C-Eri indicate samples A, B, C after enrichment pretreatment, respectively. A-7d, B-7d, and C-7d indicate samples A, B, and C after 7-day enrichment pretreatment, respectively. A-92d, B-92d, and C-92d indicate samples A, B, and C after 92-day enrichment pretreatment, respectively. Figure S4: The phylogenetic tree of strain 28AX09 based on homologous proteins. The number on the branch represents the bootstrap percentage. Figure S5: The phylogenetic tree of strain 4AJ11 based on the *16S rRNA* gene sequence. Figure S6: The phylogenetic tree of strain 4AJ11 based on homologous proteins. Figure S7: The phylogenetic tree of strain 57AJ16 based on the *16S rRNA* gene sequence. Figure S8: The phylogenetic tree of strain 57AJ16 based on homologous proteins. Figure S9: The phylogenetic tree of strain 57CJ19 based on the *16S rRNA* gene sequence. Figure S10: The phylogenetic tree of strain 57CJ19 based on homologous proteins. Figure S11: The phylogenetic tree of strain 92AX17 based on the *16S rRNA* gene sequence. Figure S12: The phylogenetic tree of strain 92AX17 based on homologous proteins. Table S1. Media components. Table S2. General information on the cultivated bacterial genera. Table S3. The best hit of 248 bacterial species cultivated based on the EzBioCloud server. Table S4. Novel candidate species isolated in this study and their degradation abilities towards chitin or cellulose. Table S5. The *p* values of ANOSIM analyses. Table S6. ANI values between the novel species and their closely related type strains.
