# Supplementary material for: Enrichment Pretreatment Expands the Microbial Diversity Cultivated from Marine Sediments
Source: Microorganisms. 2023 Nov 15;11(11):2771. doi: 10.3390/microorganisms11112771 (PMC10673404; doi:10.3390/microorganisms11112771)
Supplement: Supplementary file 1 [file microorganisms-11-02771-s001.zip › Table S1.pdf]

Table S1. Media components.

Media names	Components
Chitin solid medium	0.05% N-acetylglucosamine (w/v), 0.05% peptone (w/v), 0.01% yeast extract (w/v), 0.1% vitamin solution (v/v), 1.5% agar (w/v), fresh sea water, pH 7.5.
Cellulose solid medium	0.05% sodium carboxymethylcellulose (w/v), 0.05% peptone (w/v), 0.01% yeast extract (w/v), 0.1% vitamin solution (v/v), 1.5% agar (w/v), fresh sea water, pH 7.5.
Comprehensive solid medium	2% Monosaccharide and oligosaccharide solution (v/v), 2% sugar metabolite solution (v/v), 2% amino acid solution (v/v), 1% inorganic salt solution (v/v), 0.1% vitamin solution (v/v), 0.001% ferric chloride (w/v), 1.5% agar (w/v), fresh sea water, pH 7.5.
Chitin liquid medium	0.5% Chitin powder (w/v), 0.01% yeast extract (w/v), 0.01% ammonium chloride (w/v), 0.1% sodium nitrate (w/v), 0.1% vitamin solution (v/v), fresh sea water, pH 7.5.
Cellulose liquid medium	0.5% Filter paper cellulose (w/v), 0.01% yeast extract (w/v), 0.01% ammonium chloride (w/v), 0.1% sodium nitrate (w/v), 0.1% vitamin solution (v/v), fresh sea water, pH 7.5.
Vitamin solution	Biotin 2.0 mg/L, folate 2.0 mg/L, pyridoxine 10.0 mg/L, thiamine 5.0 mg/L, riboflavin 5.0 mg/L, niacin 5.0 mg/L, D-calcium pantothenate 5.0 mg/L, cobalamin vitamin 0.1 mg/L, p-aminobenzoic acid 5.0 mg/L, lipoic acid 5.0 mg/L. Filtration sterilization (0.22 μm), stored at -20°C.
Monosaccharide and oligosaccharide solution	Glucose 25 g/L, mannose 25 g/L, fructose 25 g/L, galactose 25 g/L, Arabinose 25 g/L, ribose 25 g/L, xylose 25 g/L, mannitol 25 g/L, N-acetylglucosamine 25 g/L, maltose 25 g/L, raffinose 25 g/L. Filtration sterilization (0.22 μm), stored at -4°C.
Sugar metabolite solution	Sodium pyruvate 25 g/L, sodium citrate 25 g/L, sodium succinate 25 g/L, sodium malate 25 g/L, sodium lactate 25 g/L, sodium acetate 25 g/L, sodium propionate 25 g/L. Filtration sterilization (0.22 μm), stored at -4°C.
Amino acid solution	Acid hydrolyzed casein 50 g/L, enzyme hydrolyzed casein 6.25 g/L, L-cysteine 1 mg/L, L-tryptophan 50 mg/L, L-methionine 50 mg/L, L-asparagine 50 mg/L, L-glutamine 50 mg/L. Filtration sterilization (0.22 μm), stored at -4°C.
Inorganic salt solution	Sodium bicarbonate 8 g/L, ammonium chloride 30 g/L, sodium sulfate 15 g/L, sodium sulfite 15 g/L, sodium nitrate 30 g/L, dipotassium hydrogen phosphate 4 g/L. Filtration sterilization (0.22 μm), stored at -4°C.
