# Supplementary figures and images for: Enrichment Pretreatment Expands the Microbial Diversity Cultivated from Marine Sediments
Source: Microorganisms. 2023 Nov 15;11(11):2771. doi: 10.3390/microorganisms11112771 (PMC10673404; doi:10.3390/microorganisms11112771)

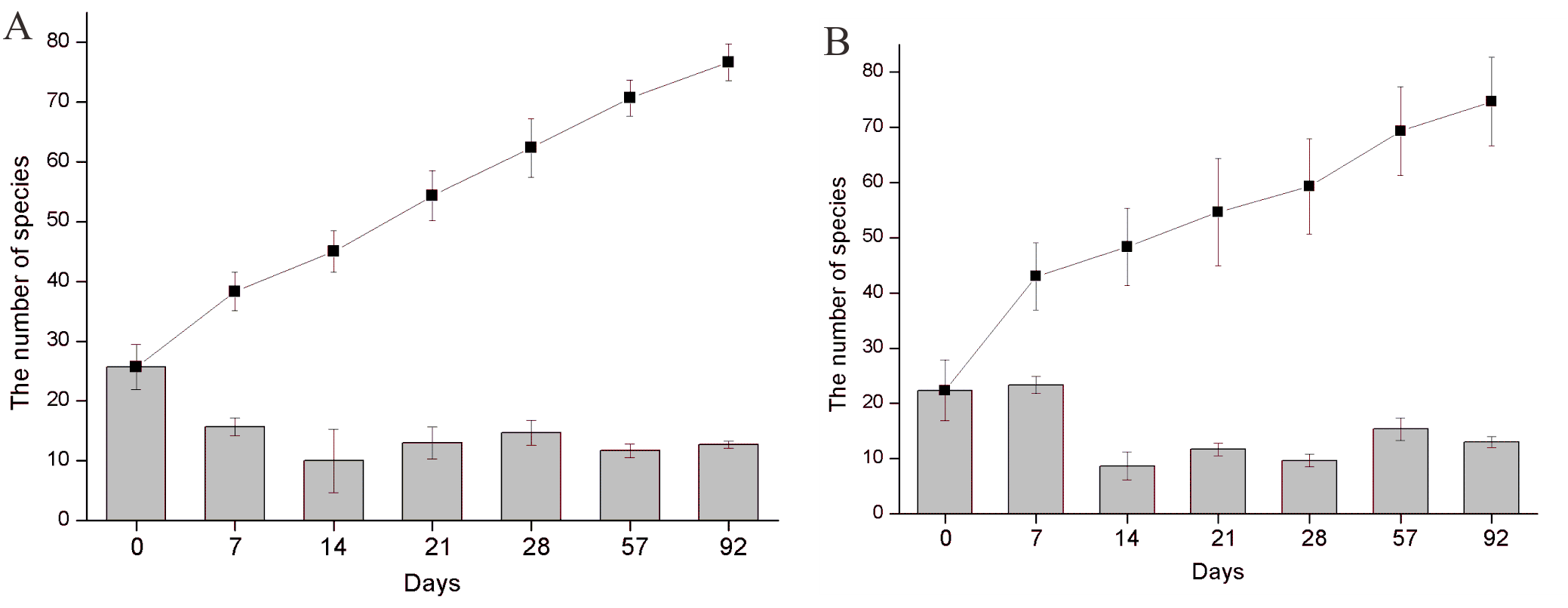

Supplement: Supplementary file 1 [file microorganisms-11-02771-s001.zip › Fig.S1.tif]

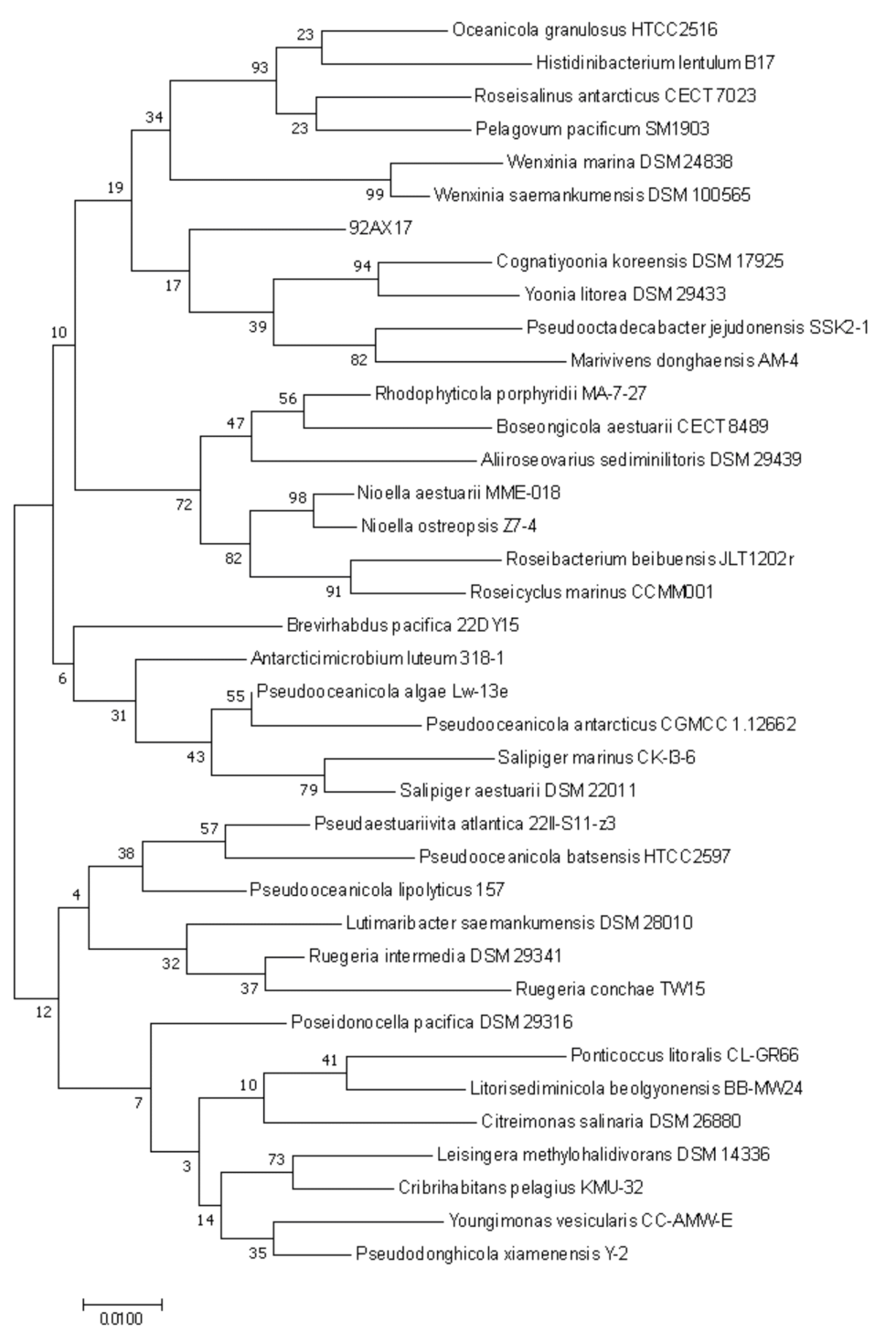

Supplement: Supplementary file 1 [file microorganisms-11-02771-s001.zip › Fig.S10.tif]

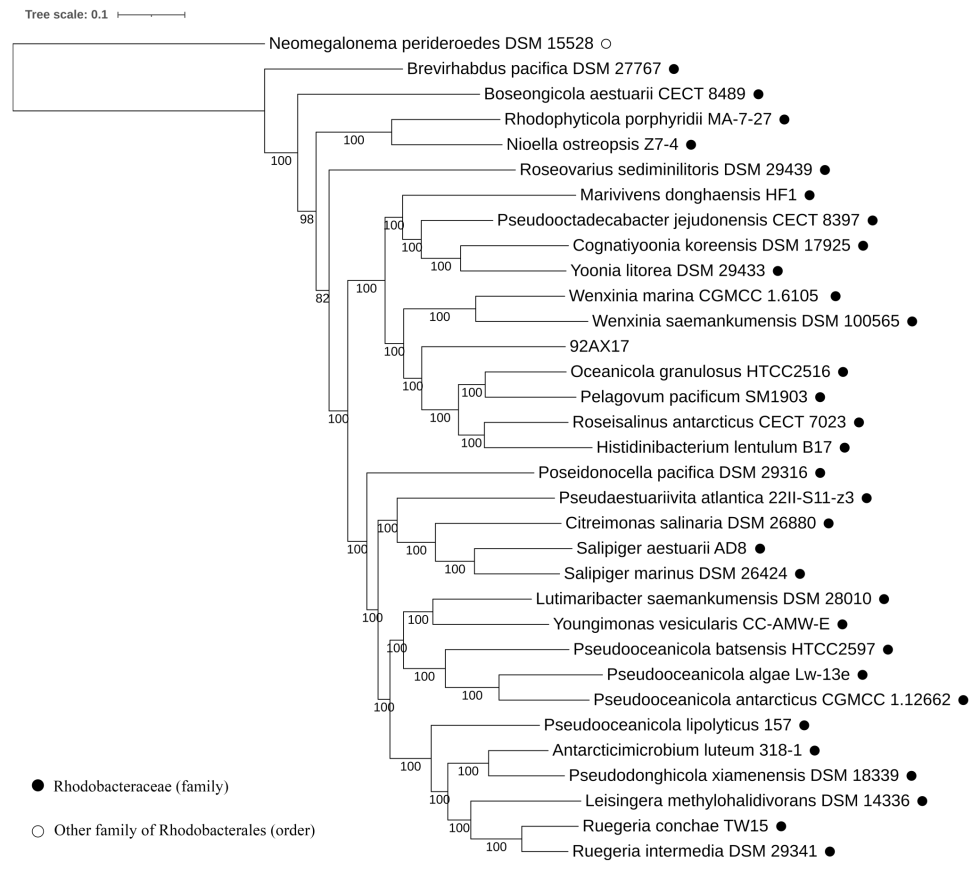

Supplement: Supplementary file 1 [file microorganisms-11-02771-s001.zip › Fig.S11.tif]

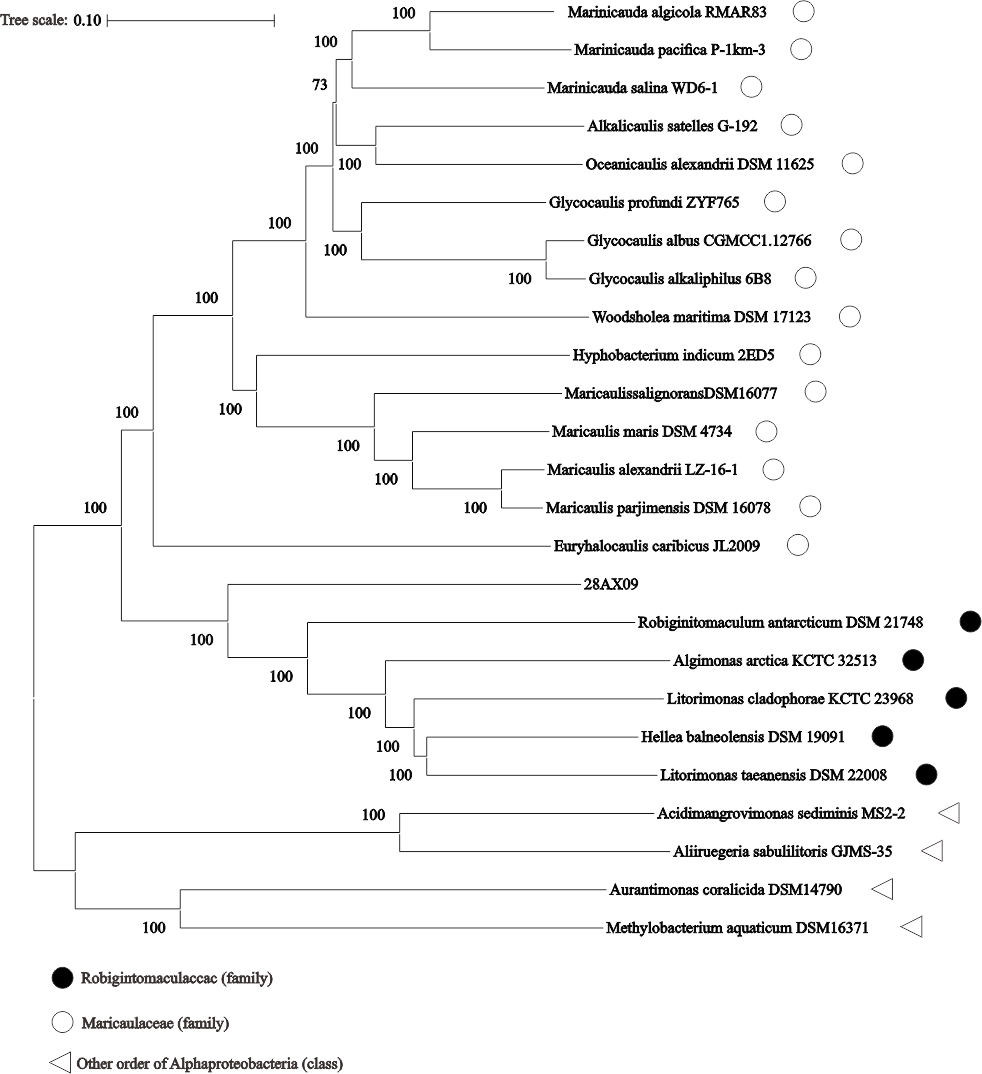

Supplement: Supplementary file 1 [file microorganisms-11-02771-s001.zip › Fig.S12.tif]

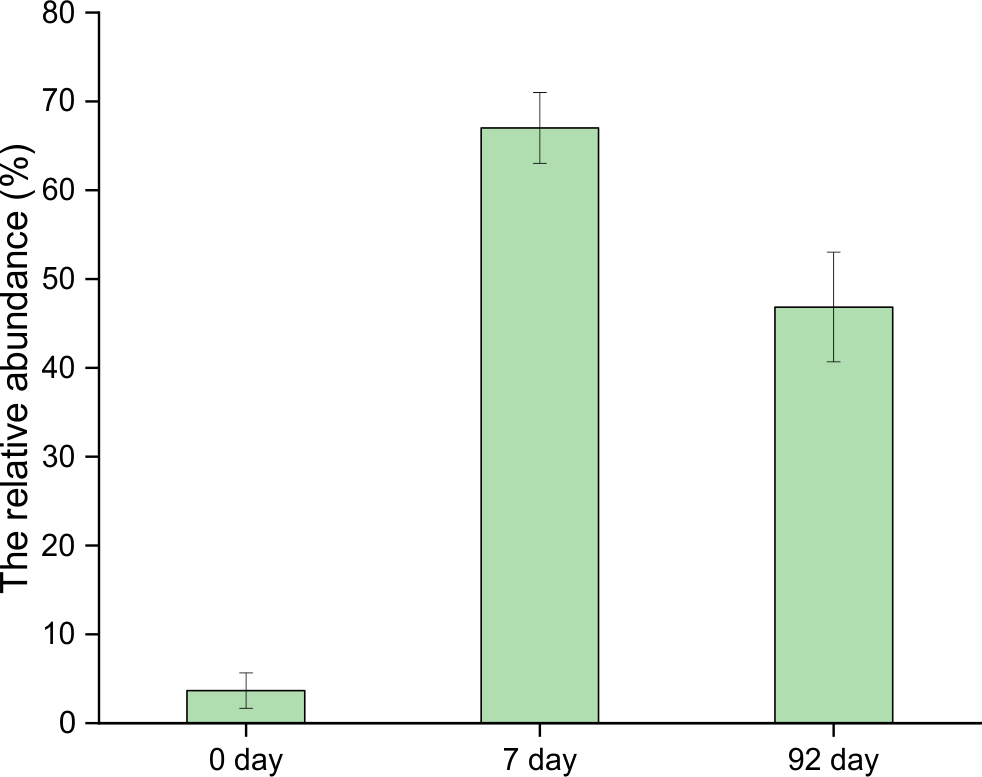

Supplement: Supplementary file 1 [file microorganisms-11-02771-s001.zip › Fig.S2.tif]

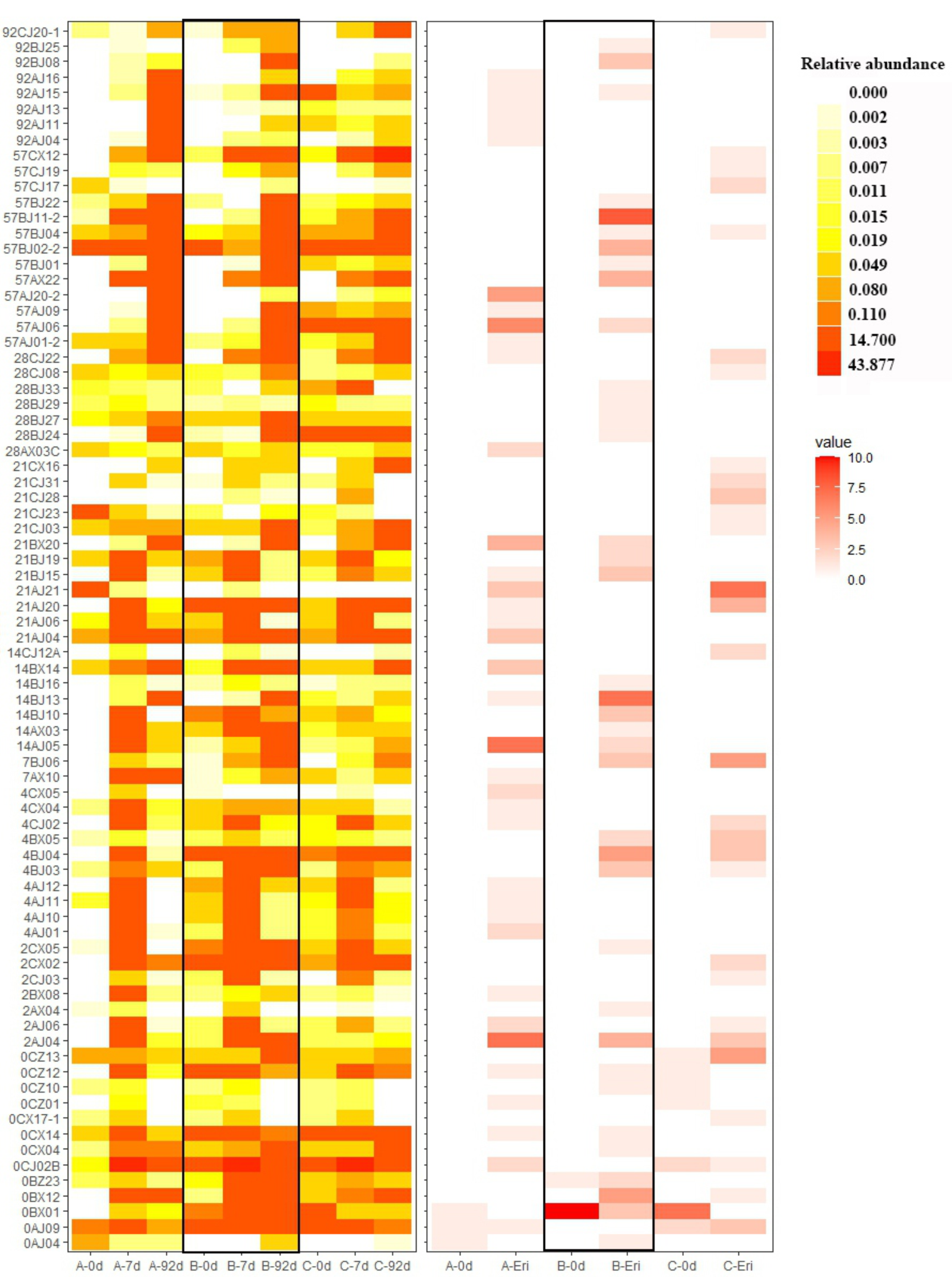

Supplement: Supplementary file 1 [file microorganisms-11-02771-s001.zip › Fig.S3.tif]

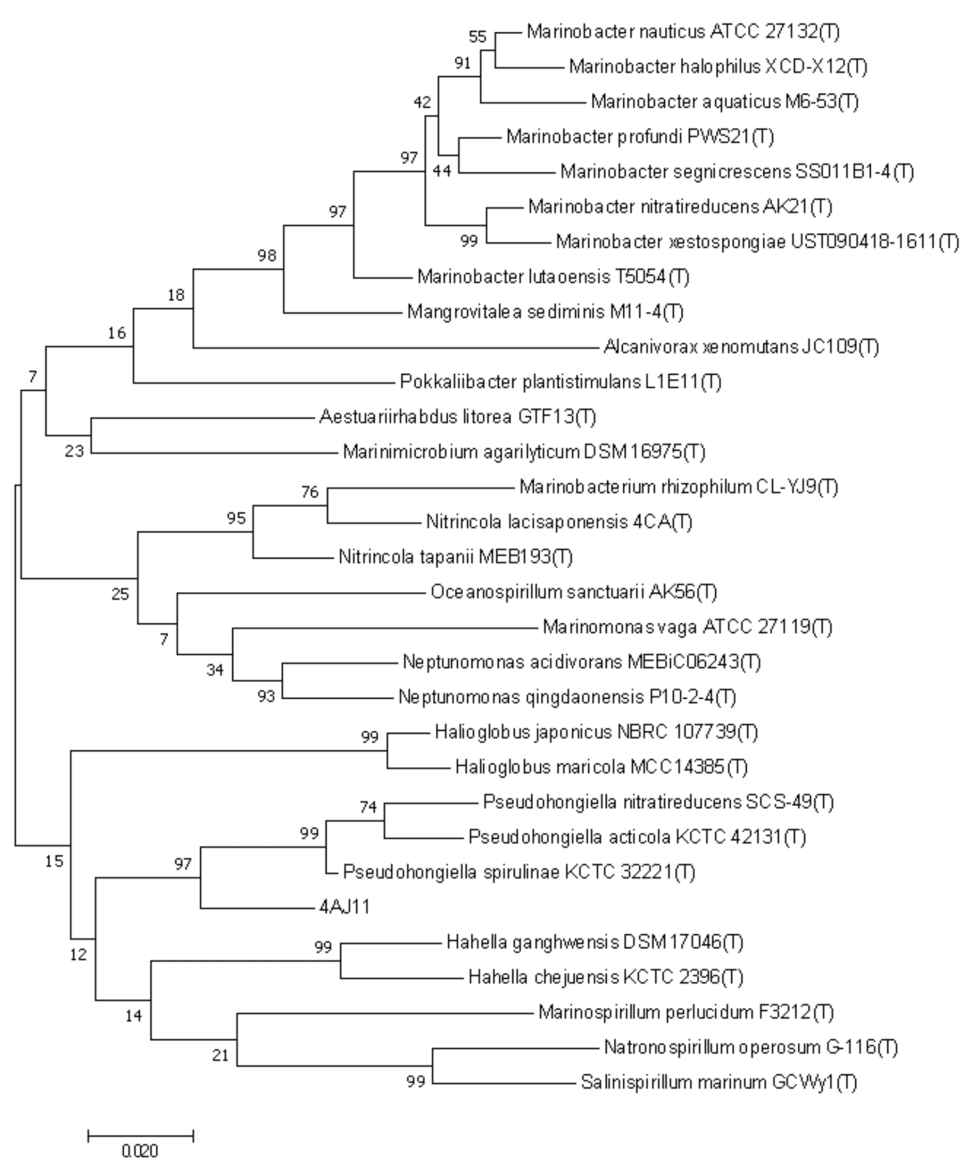

Supplement: Supplementary file 1 [file microorganisms-11-02771-s001.zip › Fig.S4.tif]

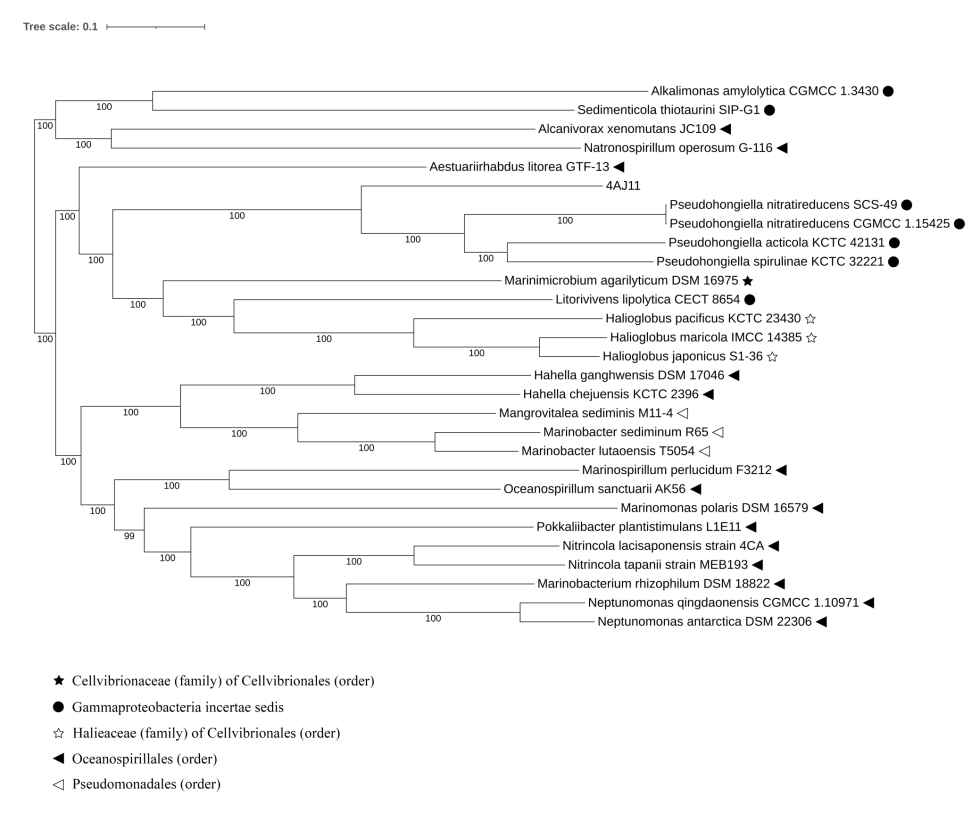

Supplement: Supplementary file 1 [file microorganisms-11-02771-s001.zip › Fig.S5.tif]

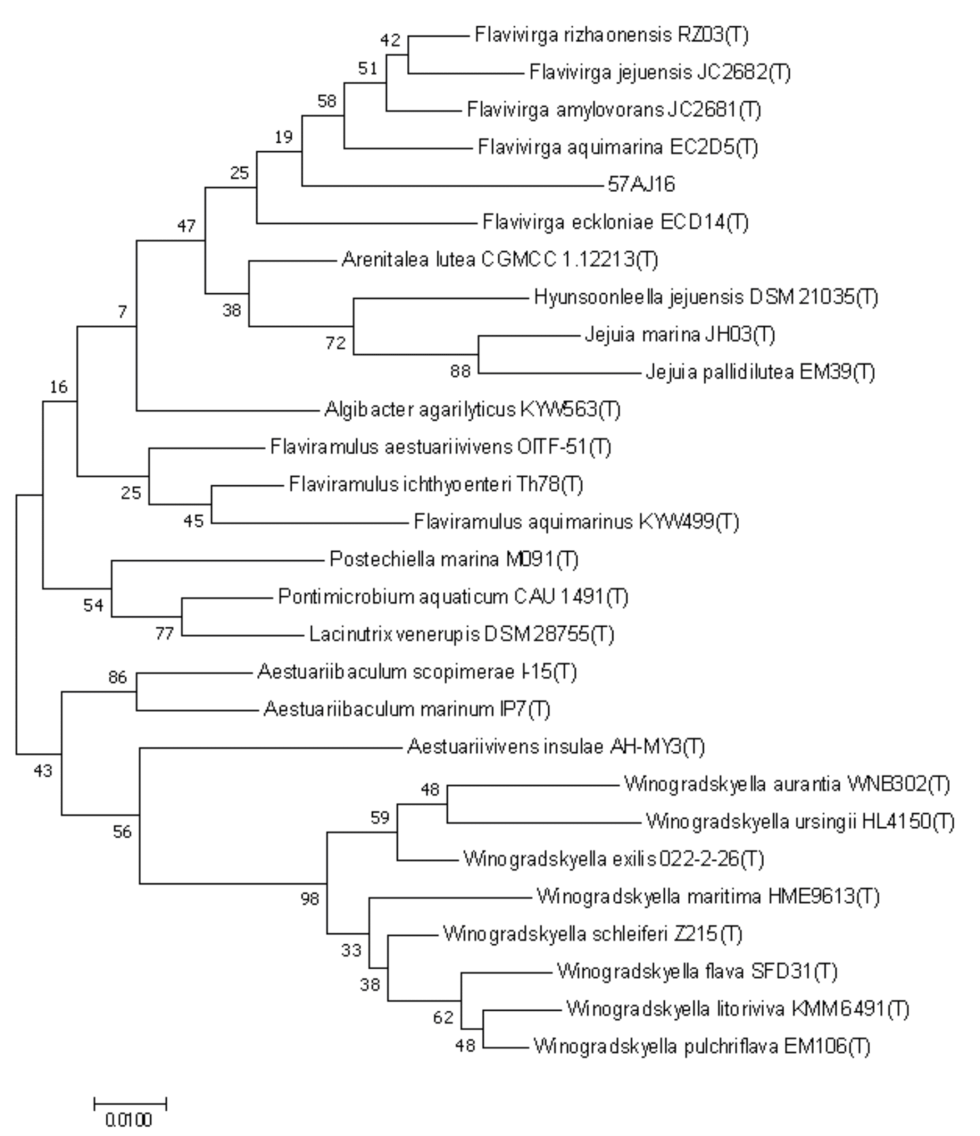

Supplement: Supplementary file 1 [file microorganisms-11-02771-s001.zip › Fig.S6.tif]

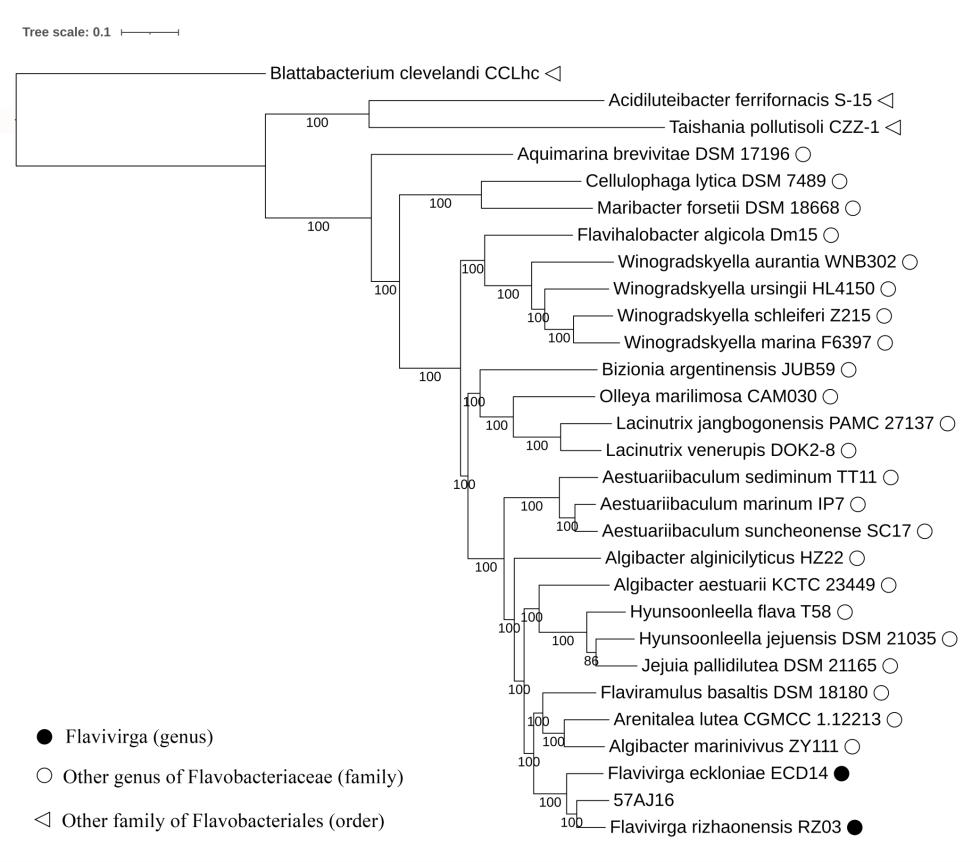

Supplement: Supplementary file 1 [file microorganisms-11-02771-s001.zip › Fig.S7.tif]

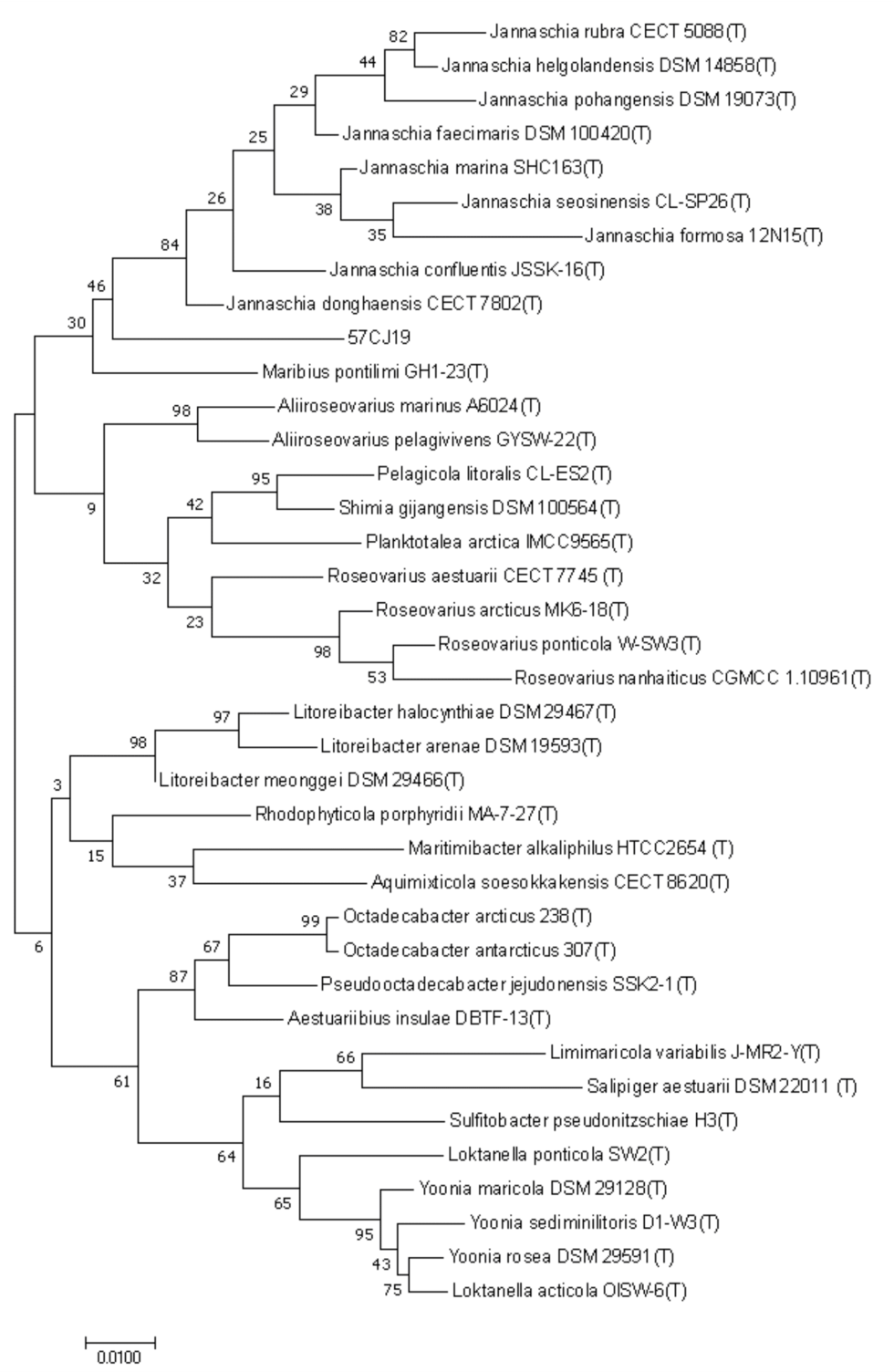

Supplement: Supplementary file 1 [file microorganisms-11-02771-s001.zip › Fig.S8.tif]

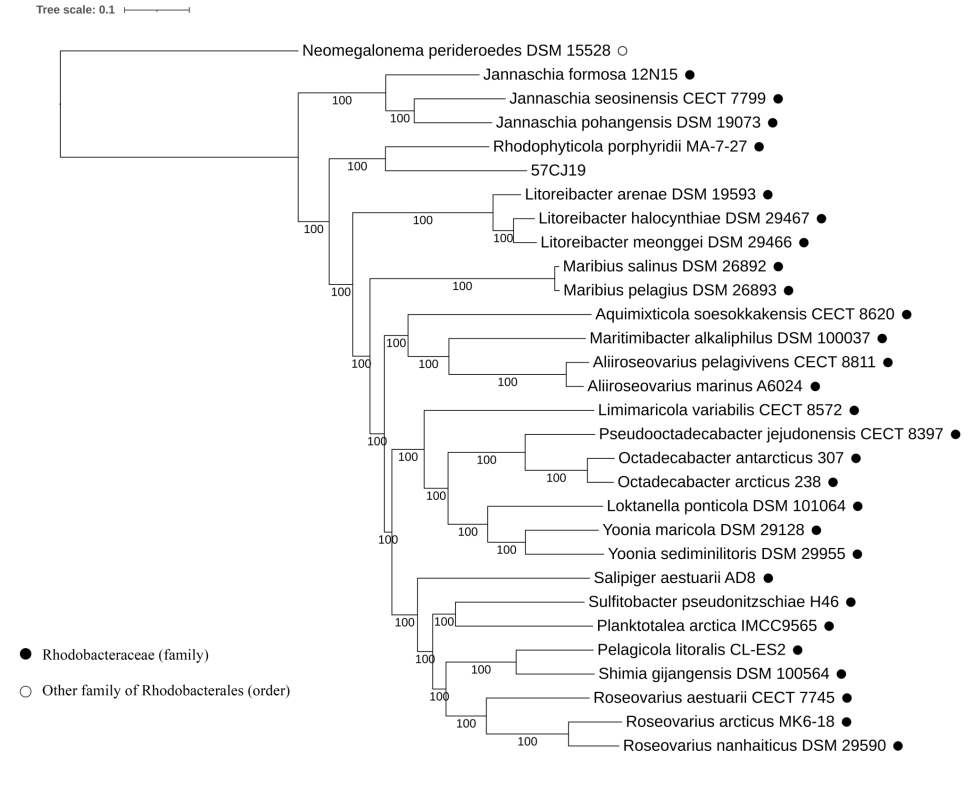

Supplement: Supplementary file 1 [file microorganisms-11-02771-s001.zip › Fig.S9.tif]
